# Consequences of extensive habitat fragmentation in landscape-level patterns of genetic diversity and structure in the Mediterranean esparto grasshopper

**DOI:** 10.1111/eva.12273

**Published:** 2015-06-01

**Authors:** Joaquín Ortego, María P Aguirre, Víctor Noguerales, Pedro J Cordero

**Affiliations:** 1 Department of Integrative Ecology, Estación Biológica de Doñana (EBD-CSIC)Seville, Spain; 2 Grupo de Investigación de la Biodiversidad Genética y Cultural, Instituto de Investigación en Recursos Cinegéticos - IREC (CSIC, UCLM, JCCM)Ciudad Real, Spain

**Keywords:** circuit theory, gene flow, genetic diversity, genetic structure, population fragmentation, population genetics, specialist species

## Abstract

Anthropogenic habitat fragmentation has altered the distribution and population sizes in many organisms worldwide. For this reason, understanding the demographic and genetic consequences of this process is necessary to predict the fate of populations and establish management practices aimed to ensure their viability. In this study, we analyse whether the spatial configuration of remnant semi-natural habitat patches within a chronically fragmented landscape has shaped the patterns of genetic diversity and structure in the habitat-specialist esparto grasshopper (*Ramburiella hispanica*). In particular, we predict that agricultural lands constitute barriers to gene flow and hypothesize that fragmentation has restricted interpopulation dispersal and reduced local levels of genetic diversity. Our results confirmed the expectation that isolation and habitat fragmentation have reduced the genetic diversity of local populations. Landscape genetic analyses based on circuit theory showed that agricultural land offers ∽1000 times more resistance to gene flow than semi-natural habitats, indicating that patterns of dispersal are constrained by the spatial configuration of remnant patches of suitable habitat. Overall, this study shows that semi-natural habitat patches act as corridors for interpopulation gene flow and should be preserved due to the disproportionately large ecological function that they provide considering their insignificant area within these human-modified landscapes.

## Introduction

Anthropogenic habitat fragmentation has altered the distribution and population sizes in many organisms worldwide and can be considered one of the major threats to biodiversity (Noss and Csuti 1994; Fahrig 2002; Lindenmayer and Fischer 2006). As a result of this process, the genetic connectivity and diversity of populations has been often negatively impacted (Frankham 1996; DiBattista 2008). Fragmentation of formerly continuous habitats can modify the dispersal routes of organisms in such a way that only suitable remnant habitats act as corridors for genetic exchange among populations (Fahrig 2007; e.g. Pavlacky et al. 2009; Jha and Kremen 2013). In turn, population subdivision and disruption of gene flow can reduce levels of genetic diversity (Frankham 1996; e.g. Levy et al. 2013; Méndez et al. 2014) and, ultimately, compromise the viability of populations and lead to local extinctions (Frankham 2005; e.g. Saccheri et al. 1998). For this reason, understanding the genetic consequences of habitat fragmentation is useful to predict the fate of populations and establish management practices aimed to preserve their evolutionary potential and ensure their long-term persistence (DiBattista 2008).

The integration of genetic and spatial data can help to determine whether postfragmentation habitat configuration has modified dispersal patterns and identify the landscape elements that most contribute to genetic connectivity (Manel et al. 2003; Storfer et al. 2007, 2010). In the absence of natural barriers to dispersal, the movement of animals is expected to be mostly determined by geographical distance in prefragmentation continuous landscapes but constrained by the spatial distribution of corridors of suitable habitat patches after fragmentation (Zellmer and Knowles 2009; Jha and Kremen 2013). However, the responses of species to landscape fragmentation are difficult to predict and highly dependent on their dispersal capacity and propensity to cross unsuitable habitat patches (Blanchet et al. 2010; DiLeo et al. 2010; Lange et al. 2010). Accordingly, some studies have found that fragmentation results in population connectivity is constrained by corridors of suitable habitat embedded within a hostile habitat matrix (e.g. Pavlacky et al. 2009; Zellmer and Knowles 2009; Jha and Kremen 2013; Ruiz-González et al. 2014), whereas others have revealed that human-driven landscape fragmentation has no effect (Quéméré et al. 2010) or even facilitates dispersal and gene flow (Bacles et al. 2005; Pavlova et al. 2012). Landscape genetics can help to identify discontinuities in gene flow and determine the relative resistance to movement imposed by different landscape elements, offering a powerful tool to understand the impacts of habitat fragmentation and inform on ground conservation practices aimed to maintain or promote population connectivity (Segelbacher et al. 2010).

The Mediterranean region has been modified over centuries of logging and land clearing for grazing and agriculture, constituting one of the areas of the world historically most altered by humans (Blondel and Aronson 1999). As a result of this process, large areas of formerly forested regions have been transformed into agricultural lands and nonfarmed areas are now often reduced to relict semi-natural habitat patches (Blondel and Aronson 1999). This is the case of La Mancha, a region from central Spain that constitutes the largest plain from the country and where land is currently devoted in its great majority to extensive cultures of wheat, barley, vineyards and olive trees. Semi-natural habitats are mostly reduced to a few scattered small hills and canyons, rock outcrops, and saline low grounds not suitable for agriculture. In this study, we analyse whether the configuration of these remnant semi-natural habitat patches within this chronically fragmented landscape has shaped the patterns of genetic diversity and structure in the habitat-specialist esparto grasshopper (*Ramburiella hispanica*) (Rambur, 1838). This Mediterranean orthoptera exclusively inhabits esparto grass formations (e.g. *Stipa tenacissima* and *Lygeum spartum*), which in La Mancha region are ubiquitous plant species in any remnant semi-natural nonagricultural land. We first characterize the habitat of the species in the study area and use circuit theory to generate different isolation-by-resistance (IBR) scenarios and test the relative importance of geographical distance and the distribution of suitable habitats on contemporary patterns of genetic differentiation (McRae 2006; McRae and Beier 2007; McRae et al. 2008). Second, we examine whether habitat fragmentation has negatively impacted local levels of genetic diversity (Frankham 1996; e.g. Méndez et al. 2014; Levy et al. 2013). In particular, we (i) predict that agricultural lands constitute barriers to gene flow and (ii) hypothesize that fragmentation has restricted interpopulation dispersal and reduced local effective population sizes, resulting in populations located in more fragmented and isolated habitat patches have lower levels of genetic diversity.

## Materials and methods

### Study sites and sampling

**Figure 1:**
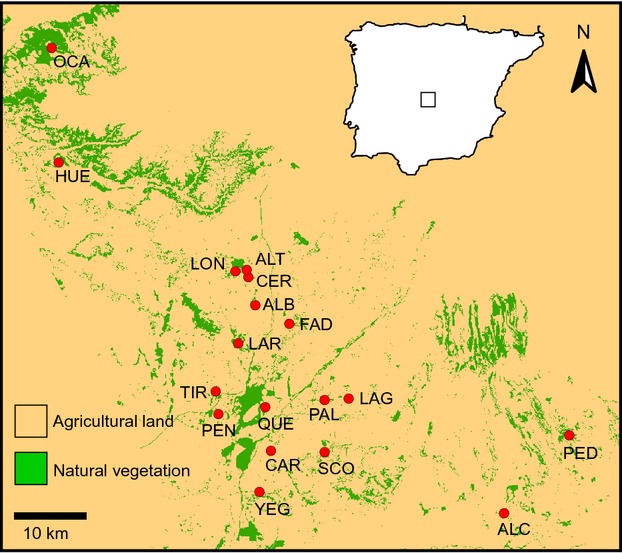
Geographical location of the studied populations of esparto grasshopper (*Ramburiella hispanica*) (red dots) and land-cover types classified as ‘natural vegetation’ (noncultivated areas adequate for the species) and ‘agricultural land’ (mostly cultivated areas and, in a much lesser extent, lagoons, reservoirs, villages and other developed areas). Population codes are described in Table1.

During 2010, we sampled 352 individuals from 18 populations of esparto grasshoppers in La Mancha region, central Spain (∽2500 km^2^; Fig.1). We aimed to sample 20 specimens and an equal number of males and females in each locality, but samples sizes are often male-biased due to the difficulties to capture females in some sites (Table1). Specimens were preserved whole in 1500 *μ*L of 96% ethanol and stored at −20°C until needed for genetic analyses. Population code descriptions and further information on sampling localities are given in Table1 and Fig.1.

**Table 1:** Geographical location, sample size (number of males/females in parentheses) and genetic diversity (*A*_R_: standardized allelic richness; *H*_E_: gene diversity) for each study population of *Ramburiella hispanica* in La Mancha region

Locality	Code	Latitude	Longitude	*N*	*A*_R_	*H*_E_
Saladar de Ocaña	OCA	39.985445	−3.630508	20 (12/8)	10.32	0.864
Huerta de Valdecarábanos	HUE	39.838697	−3.617103	18 (15/3)	10.48	0.867
Laguna de El Cerrillo	CER	39.694744	−3.301181	20 (10/10)	9.97	0.857
Laguna de El Altillo	ALT	39.703076	−3.302290	20 (10/10)	10.24	0.848
Laguna de Longar	LON	39.700548	−3.321046	20 (10/10)	10.46	0.852
Laguna de La Albardiosa	ALB	39.658024	−3.288700	20 (10/10)	9.64	0.845
Villa de Don Fadrique	FAD	39.634933	−3.231576	20 (10/10)	8.86	0.821
Laguna Larga	LAR	39.609088	−3.317164	19 (11/8)	10.65	0.861
Laguna de Tírez	TIR	39.546603	−3.354411	19 (9/10)	10.39	0.857
Laguna de Peña Hueca	PEN	39.517720	−3.350181	19 (10/9)	10.14	0.859
Laguna de Quero	QUE	39.526984	−3.272089	20 (9/11)	10.21	0.865
Laguna de Los Carros	CAR	39.472016	−3.262528	19 (9/10)	10.41	0.854
Laguna de Las Yeguas	YEG	39.418396	−3.281576	20 (10/10)	10.56	0.860
Laguna de Palomares	PAL	39.535906	−3.172344	20 (10/10)	10.44	0.864
Laguna de La Laguna	LAG	39.538542	−3.134392	19 (10/9)	9.57	0.835
Laguna de Salicor	SCO	39.470083	−3.173809	20 (14/6)	10.86	0.864
Saladar de El Pedernoso	PED	39.491164	−2.767518	20 (17/3)	11.12	0.881
Laguna de Alcahozo	ALC	39.391585	−2.875947	19 (15/4)	9.04	0.840

### Microsatellite genotyping and basic genetic statistics

We used a salt extraction protocol to purify genomic DNA from a hind leg of each individual (Aljanabi and Martinez 1997). We used 12 highly polymorphic microsatellite markers to genotype each sampled individual (Aguirre et al. 2014; Table S1). Amplifications were conducted in 10-*μ*L reaction volumes containing approximately 20 ng of template DNA, 1× reaction buffer (67 mm Tris–HCL, pH 8.3, 16 mm (NH_4_)_2_SO_4_, 0.01% Tween-20, EcoStart Reaction Buffer; Ecogen, Madrid, Spain), 2 mm MgCl_2_, 0.2 mm of each dNTP, 0.15 *μ*m of each dye-labelled primer (FAM, PET, VIC or NED) and 0.1 U of *Taq* DNA EcoStart Polymerase (Ecogen). The PCR programme used was 9 min denaturing at 95°C followed by 40 cycles of 30 s at 94°C, 45 s at the annealing temperature (Table S1) and 45 s at 72°C, ending with a 10-min final elongation stage at 72°C. Amplification products were electrophoresed using an ABI 310 Genetic Analyzer (Applied Biosystems, Foster City, CA, USA), and genotypes were scored using GeneMapper 3.7 (Applied Biosystems).

Microsatellite loci were tested for departure from Hardy–Weinberg equilibrium within each sampling population using an exact test (Guo and Thompson 1992) based on 900 000 Markov chain iterations as implemented in the program Arlequin 3.1 (Excoffier et al. 2005). We also used Arlequin 3.1 to test for linkage disequilibrium between each pair of loci for each sampling population using a likelihood-ratio statistic, whose distribution was obtained by a permutation procedure (Excoffier et al. 2005). We applied sequential Bonferroni corrections to account for multiple comparisons (Rice 1989).

### GIS analyses

In our study area, the esparto grasshopper distributes in areas with formations of the host plants *S. tenacissima* and *L. spartum*, which are ubiquitous plant species present in any noncultivated habitat patch from La Mancha region. The esparto grasshopper has never been recorded in agricultural lands or developed areas (Pardo and Gómez 1995 and references therein). For this reason, we classified our study area in two landscape element classes: ‘natural vegetation’ (noncultivated semi-natural areas optimal for the species) and ‘agricultural land’ (mostly cultivated areas and, in a much lesser extent, lagoons, reservoirs, villages and other developed areas unsuitable for the species). This information was obtained by digitalizing habitat patches from the most recent aerial pictures available at Centro Nacional de Información Geográfica from Spain (http://centrodedescargas.cnig.es/CentroDescargas/). We used ArcMap 10.0 (ESRI, Redlands, CA, USA) to create a vector layer that was then transformed into a raster grid (pixel size = 50 m) to be used in subsequent landscape genetic analyses (see below).

### Genetic diversity

For each population, we calculated allelic richness (*A*_R_) standardized for sample size using the program HP-Rare (Kalinowski 2005) and gene diversity (*H*_E_) using *F*_STAT_ (Goudet 1995). We used an information-theoretic model-selection approach to analyse which variables contribute to explain patterns of genetic diversity (*A*_R_ and *H*_E_) across the studied populations of esparto grasshopper. We considered four explanatory variables: (i) the proportion of suitable habitat (i.e. ‘natural vegetation’; see previous section) within a 1 km radius of the sampling site, which we hypothesize to be associated with local effective population sizes (*N*_e_) (see also Levy et al. 2013 for a similar approach); (ii) average genetic differentiation (*F*_ST_) of each population with all other populations, an estimate of population isolation (e.g. Wang et al. 2011; Ortego et al. 2012); (iii) latitude; and (iv) longitude. We used general linear models (GLM) with a normal error structure and identity link function as implemented in the R 3.0.0 package lme4 (R Core Team 2012). The precision of *A*_R_ estimates may differ among populations due to small differences in sample sizes, and we took this into account using a weighted least square method, where weight equals the sample size for each studied population. We ranked the resulting models following a model-selection approach on the basis of the Akaike's information criterion corrected for small sample size (AIC_c_; Burnham and Anderson 1998). AIC_c_ values for each model were rescaled (ΔAIC_c_) calculating the difference between the AIC_c_ value of each model and the minimum AIC_c_ obtained among all competing models (i.e. the best model has ΔAIC_c_ = 0). Models with ΔAIC_c_ ≤ 2 were considered equivalent (Burnham and Anderson 1998). In cases where model selection as a function of AIC_c_ did not give a single model, we performed an averaging of equivalent models (i.e. with ΔAICc ≤ 2; Burnham and Anderson 2002). We calculated the mean of the predictor estimators, their unconditional standard errors (SE) and confidence intervals (CIs), and the relative importance of each variable in the final averaged model (Σ *ω*i, the sum of Akaike weights of models with ΔAIC_c_ ≤ 2 in which the variable was included). The effect of predictor variables was considered significant if the 95% CI of their estimators did not cross zero (Burnham and Anderson 2002). Model selection and averaging was performed using the R package AICcmodavg (R Core Team 2012).

### Genetic structure

We estimated genetic differentiation between populations calculating pairwise *F*_ST_ values and testing their significance with Fisher's exact tests after 10 000 permutations as implemented in Arlequin 3.1 (Excoffier et al. 2005). Critical *P*-values for pairwise tests of allelic differentiation were determined using a sequential Bonferroni adjustment (Rice 1989). Due to the frequent presence of null alleles in grasshoppers (e.g. Chapuis et al. 2008; Blanchet et al. 2012a), we used the program FreeNA to estimate null allele frequencies and calculate pairwise *F*_ST_ values corrected for null alleles using the so-called ENA method (Chapuis and Estoup 2007). We used the randomization method implemented in *F*_STAT_ 2.9.3 (10 000 permutations) to analyse differences between males and females in interpopulation genetic differentiation (*F*_ST_), deviation from Hardy–Weinberg equilibrium (*F*_IS_), mean assignment index (mAIc) and variance of the assignment index (vAIc) (Goudet et al. 2002), parameters that are informative about sex-biased patterns of dispersal (e.g. Ortego et al. 2011). Finally, we analysed genetic structure using the Bayesian clustering analysis implemented in the program Tess 2.3.1, which incorporates geographical coordinates as *a priori* information (Chen et al. 2007; Durand et al. 2009). We ran Tess 2.3.1 under the conditional autoregressive (CAR) Gaussian model of admixture with a linear trend surface (Durand et al. 2009), which is expected to be more robust against overestimation of *K*_max_ in the presence of genetic clines (Guillot 2009; Francois and Durand 2010). We conducted 20 independent replicates for each value of *K* = 2–12 using 50 000 sweeps and a burn-in period of 10 000 sweeps. The admixture parameter (*α*) and the interaction parameter (*w*) were initially set to *α *= 0.99 and *w* = 0.6. We used deviance information criterion (DIC) values and stabilization of the *Q*-matrix of posterior probabilities to determine the optimal number of clusters (i.e. *K*_max_) for the data. Once *K*_max_ was deduced, 180 additional replicate runs were conducted to yield a total of 200 replicate runs for *K*_max_. We used the 10 runs with the lowest DIC values to calculate average individual admixture proportions with Clumpp 1.1.2 (Jakobsson and Rosenberg 2007), which were visualized as a bar plot using Distruct 1.1 (Rosenberg 2004).

### Landscape genetic analyses

We applied circuit theory to model gene flow across a spatially heterogeneous landscape and determine the impact of isolation-by-distance (IBD) and different IBR scenarios on observed patterns of genetic differentiation (McRae 2006; e.g. McRae and Beier 2007). We used Circuitscape 3.5.8 to calculate resistance distance matrices between all pairs of populations considering an eight-neighbour cell connection scheme (McRae 2006). We used the raster layer obtained as described in the section ‘*GIS analyses*’, which includes the two landscape element classes (‘natural vegetation’ and ‘agricultural land’) that *a priori* are the most likely to determine the distribution and dispersal patterns in our study species. We generated different IBR scenarios assigning a range of resistance values to both habitat classes (Table2). This allowed us to identify the optimal ratio of landscape resistance between ‘natural vegetation’ and ‘agricultural land’ habitat classes that best fit our data of genetic differentiation (e.g. Andrew et al. 2012; Seymour et al. 2013). To test the effect of IBD, we calculated a matrix of Euclidean geographical distances between sampled populations using Geographic Distance Matrix Generator 1.2.3 (Ersts 2011). We also generated a matrix of resistances in Circuitscape considering an entirely ‘flat’ landscape, that is based on a raster layer in which all cells have equal resistance (resistance = 1). This matrix of flat resistance distances is expected to yield similar results than the matrix of Euclidean geographical distances, but the former has been suggested to be more appropriate for comparison with models of IBR generated with Circuitscape (Lee-Yaw et al. 2009; Munshi-South 2012; Jha and Kremen 2013). Geographical distance and IBR matrices were tested against matrices of pairwise *F*_ST_ values using a multiple matrix regression with randomization (MMRR) approach (Wang 2013). We used the MMRR function script implemented in R 3.0.2 (Wang 2013). Finally, we determined how well data on pairwise genetic differentiation fit the different IBR models (using the coefficient of determination, *R*^2^) with varying levels of ‘natural vegetation’/‘agricultural land’ resistance ratios (e.g. Andrew et al. 2012).

**Table 2:** Multiple Matrix Regressions with Randomization (MMRR) for genetic differentiation (*F*_ST_ and *F*_ST_ corrected for null alleles) in relation with geographical distance (isolation-by-distance, IBD) and 21 isolation-by-resistance (IBR) scenarios considering different resistance values (1 = lowest resistance; 100 000 = highest resistance) for the two land-cover types considered in this study (‘natural vegetation’ and ‘agricultural land’)

Model	Natural	Agricultural	Ratio	*F*_ST_				*F*_ST_ corrected for null alleles			
				*R* ^2^	*β*	*t*	*P*	*R* ^2^	*β*	*t*	*P*
IBD	–	–	–	0.025	0.149	1.95	0.405	0.001	−0.010	−0.05	0.988
IBR-A	1	1	1	0.019	0.186	1.72	0.510	0.001	−0.030	−0.32	0.889
IBR-B	100	1	0.01	0.068	−0.243	−3.33	0.210	0.153	−0.358	−5.22	0.059
IBR-C	50	1	0.02	0.063	−0.235	−3.19	0.253	0.071	−0.295	−3.39	0.200
IBR-D	25	1	0.04	0.054	−0.222	−2.95	0.300	0.026	−0.201	−2.03	0.445
IBR-E	100	25	0.25	0.011	−0.118	−1.30	0.637	0.147	−0.355	−5.11	0.057
IBR-F	100	50	0.5	0.000	0.007	0.07	0.981	0.026	−0.201	−2.03	0.417
IBR-G	50	25	0.5	0.000	0.007	0.07	0.978	0.011	0.136	1.27	0.598
IBR-H	50	100	2	0.063	0.336	3.18	0.183	0.138	−0.349	−4.92	0.083
IBR-I	25	50	2	0.063	0.336	3.18	0.151	0.011	0.136	1.27	0.606
IBR-J	25	100	4	0.106	0.422	4.22	0.057	0.039	0.253	2.48	0.282
IBR-K	1	25	25	0.173	0.494	5.63	0.020	0.115	0.397	4.43	0.069
IBR-L	1	50	50	0.188	0.503	5.91	0.018	0.139	0.428	4.94	0.049
IBR-M	1	100	100	0.198	0.510	6.11	0.015	0.162	0.454	5.40	0.033
IBR-N	1	500	500	0.211	0.513	6.36	0.015	0.202	0.495	6.18	0.017
IBR-O	1	1000	1000	0.212	0.511	6.38	0.017	0.212	0.504	6.37	0.019
IBR-P	1	2000	2000	0.212	0.508	6.38	0.017	0.218	0.509	6.49	0.019
IBR-Q	1	5000	5000	0.211	0.505	6.36	0.015	0.223	0.512	6.57	0.016
IBR-R	1	10 000	10 000	0.211	0.504	6.35	0.011	0.224	0.513	6.60	0.012
IBR-S	1	20 000	20 000	0.211	0.503	6.35	0.013	0.225	0.513	6.62	0.017
IBR-T	1	50 000	50 000	0.210	0.502	6.34	0.015	0.225	0.513	6.63	0.011
IBR-U	1	100 000	100 000	0.210	0.502	6.34	0.011	0.226	0.513	6.63	0.009

## Results

### Microsatellite data

All microsatellite markers were highly polymorphic across all populations, with 12–40 alleles per locus (Table S1). After applying sequential Bonferroni corrections to compensate for multiple statistical tests, two loci (RhA113 and RhC1) consistently deviated from HWE across all the studied populations and were excluded from further analyses (Table S1). The frequency of null alleles in the different loci ranged from moderately low (<0.10; RhA108, RhB107, RhC112, RhD2 and RhB2) to high (**≥**0.20; RhA105, RhA112, RhA2 and RhC2) values (Table S1). We did not find any evidence of genotypic linkage disequilibrium at any pair of loci in any population (exact tests; all *P*s > 0.05).

### Genetic diversity

**Figure 2:**
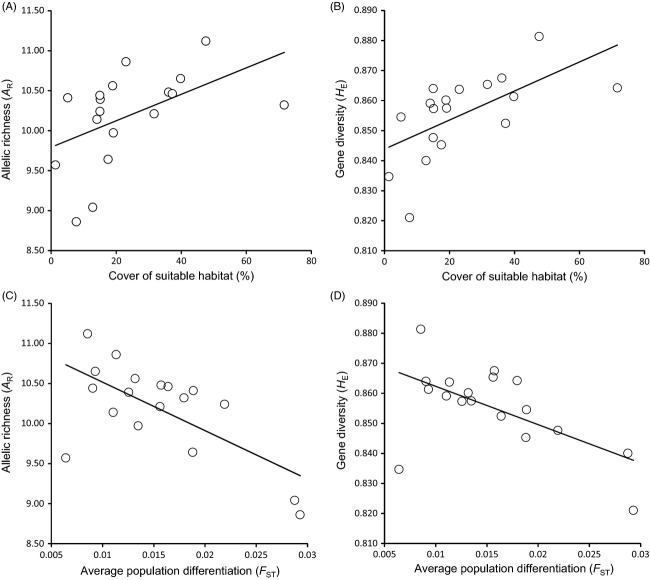
Consequences of habitat loss and population subdivision on the genetic diversity of the studied populations of esparto grasshopper (*Ramburiella hispanica*). Panels show (A, C) standardized allelic richness (*A*_R_) and (B, D) gene diversity (*H*_E_) in relation with (A, B) cover of suitable habitat (%) and (C, D) average genetic differentiation with other populations (*F*_ST_).

*A*_R_ and *H*_E_ for each population are indicated in Table1. Our most parsimonious models revealed that both *A*_R_ and *H*_E_ increased significantly with the proportion of suitable habitat and decreased with isolation estimated as average genetic differentiation (*F*_ST_) with other populations (Table S2 and Table3; Fig.2). All other tested models for *A*_R_ had a ΔAIC_c_ value > 2 (Table S2). For *H*_E_, the model also including longitude was equivalent to the best ranked model (ΔAIC_c_ value = 0.22; Table S2). However, unconditional CIs for longitude crossed zero, indicating that this variable had no significant effect (Table3).

**Table 3:** General linear models (GLMs) for (a) standardized allelic richness (*A*_R_) and (b) gene diversity (*H*_E_). A single model with ΔAICc ≤ 2 was obtained for *A*_R_. For *H*_E_, we performed model averaging of the two best ranked equivalent models (ΔAICc ≤ 2) to obtain parameter estimates and unconditional standard errors (S.E.) (see Table S2 in Supporting information). The relative importance of each variable is indicated (Σ *ω*i, sum of Akaike weights of models with ΔAICc ≤ 2 in which the variable was present). Bold type indicates significant variables, that is variables for which their unconditional 95% confidence interval (CI) did not cross zero

	Estimate ± SE	Σ *ω*i	Upper 95% CI	Lower 95% CI
(a) Allelic richness (*A*_R_)				
Intercept	10.73 ± 0.30			
Cover of suitable habitat	1.33 ± 0.55	0.51	**0.25**	**2.42**
Average population differentiation	−55.71 ± 14.99	0.51	−**85.08**	−**26.33**
(b) Gene diversity (*H*_E_)				
Intercept	0.86 ± 0.01			
Cover of suitable habitat	0.04 ± 0.01	0.78	**0.02**	**0.07**
Average population differentiation	−1.13 ± 0.34	0.78	−**1.79**	−**0.47**
Longitude	0.01 ± 0.01	0.37	−0.01	0.03

### Genetic structure

**Figure 3:**
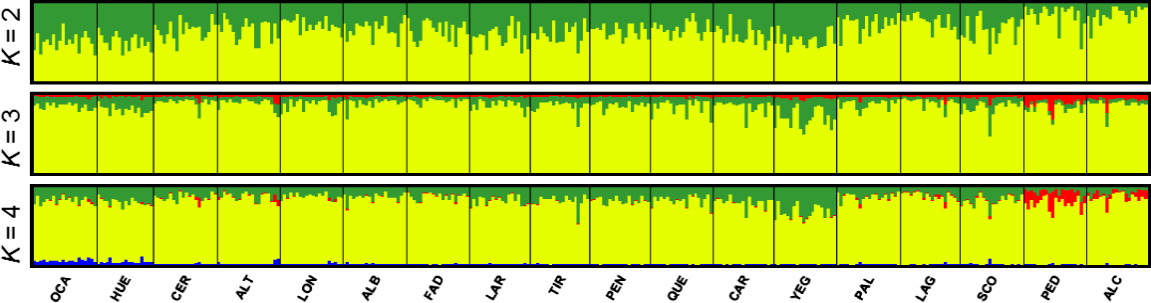
Genetic structure of the studied populations of esparto grasshopper (*Ramburiella hispanica*). Figure shows the genetic assignment based on the Bayesian method implemented in the program Tess for different numbers of genetic clusters (*K*). Each individual is represented by a thin vertical line, which is partitioned into *K*-coloured segments that represent the individual's probability of belonging to the cluster with that colour. Black lines separate individuals from different populations. Population codes are described in Table1.

Pairwise *F*_ST_ values ranged from 0.0002 to 0.0469, and 73 of the 153 pairwise comparisons were significant after sequential Bonferroni correction (Table S3). The randomization method implemented in *F*_STAT_ showed that genetic differentiation (*F*_ST_) (*P* = 0.671), deviation from Hardy–Weinberg equilibrium (*F*_IS_) (*P *=* *0.586), mAIc (*P *=* *0.959) and vAIc (*P *=* *0.348) did not differ between males and females. Individual-based analyses in Tess resulted in a *K*_max_ = 4, but three genetic clusters were scarcely represented in most populations (Fig.3; Table S4).

### Landscape genetic analyses

**Figure 4:**
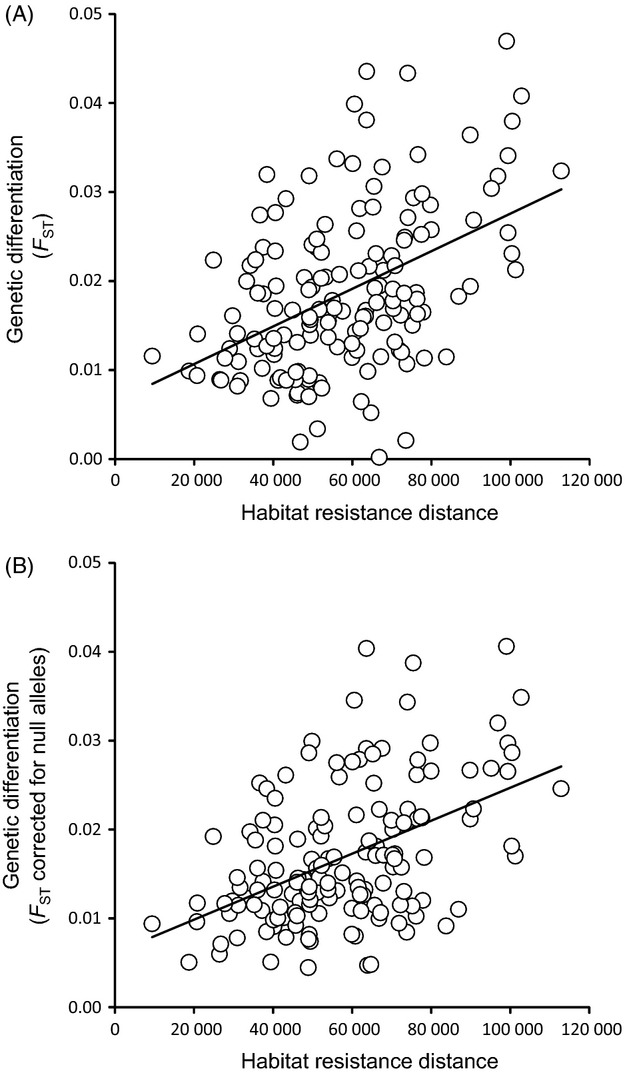
Relationship between genetic differentiation and landscape resistance distances in the studied populations of esparto grasshopper (*Ramburiella hispanica*). Genetic differentiation (panel A: *F*_ST_; panel B: *F*_ST_ corrected for null alleles) is plotted against resistance distances calculated using Circuitscape considering a resistance ratio of 100 000:1 for ‘agricultural land’/’natural vegetation’.

**Figure 5:**
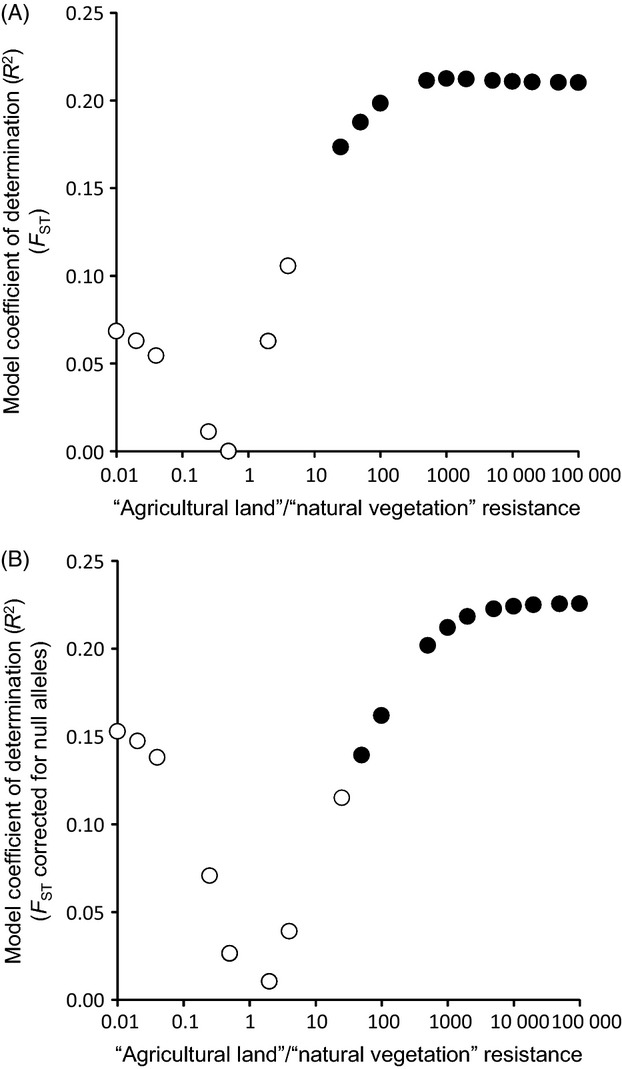
Goodness of fit for models of landscape resistance considering different resistance ratios for agricultural land and natural vegetation. Panels show the coefficient of determination (*R*^2^) for models analysing genetic differentiation (panel A: *F*_ST_; panel B: *F*_ST_ corrected for null alleles) in relation to isolation-by-resistance (IBR) distance matrices plotted against resistance ratios for ‘agricultural land’ and ‘natural vegetation’ used to calculate resistance-based distances with Circuitscape. Resistance ratios for both habitat classes are log-transformed for illustrative purposes. Filled dots indicate significant models.

Euclidean geographical distances (IBD) and flat resistance distances were highly correlated (*r *=* *0.99, *P *<* *0.0001) and they were not significantly associated with genetic differentiation, either when they were included alone in the model (Table2; Fig.4A) or together with any IBR matrix (all *P*s > 0.2). However, genetic differentiation was positively and significantly associated with those IBR matrices considering the minimum resistance for ‘natural vegetation’ habitat (=1) and high resistance values for ‘agricultural land’ (>50) (Table2; Fig.4B). Model fit increased with the ‘agricultural land’/’natural vegetation’ resistance ratio, but the strength of the relationship between genetic differentiation and IBR stabilized beyond a ratio 1000:1 (Table2; Fig.5). Analyses based on *F*_ST_ corrected for null alleles gave very similar results, but best models reached slightly higher values of *R*^2^ (Fig.5B; Table2). Further analyses based on *F*_ST_ values calculated only considering the five loci with low frequencies of null alleles (RhA108, RhB107, RhC112, RhD2 and RhB2; Table S1) provided analogous results (not shown), indicating that the effects of null alleles on the obtained results are minimal (e.g. Phillipsen et al. 2015).

## Discussion

Our results confirmed our original expectations that landscape configuration has impacted gene flow and genetic diversity of esparto grasshopper, indicating that historical fragmentation of natural habitats as a result of land clearing for agriculture has shaped its dispersal patterns and local effective population sizes. Analyses of spatial patterns of genetic structure showed the presence of a shallow genetic differentiation in this species, with a high degree of genetic admixture and low *F*_ST_ values (Fig.3; Table S3). The observed patterns of genetic structure in the esparto grasshopper contrast with those reported in other specialist grasshoppers showing deep genetic structures at similar or much shorter spatial scales (Ortego et al. 2011, 2012; Keller et al. 2013a) and can be considered comparable to the levels of genetic differentiation found in widespread generalist orthopterans that are likely to be only moderately impacted by habitat fragmentation (Wiesner et al. 2011; Blanchet et al. 2012b; Keller et al. 2013b). The esparto grasshopper is a specialist species that exclusively inhabits *S. tenacissima* and *L. spartum* grass formations in the study area. However, these host plant species are ubiquitous in any semi-natural habitat patch not devoted to agriculture, which may have contributed to maintain moderately high levels of gene flow within the study area despite a considerable landscape fragmentation. This contrasts with the scenario faced by the co-distributed grasshopper *Mioscirtus wagneri*, a habitat specialist with low dispersal capacity exclusively depending on the plant *Suaeda vera* for feeding (Ortego et al. 2010, 2012). This plant exclusively grows in saline low grounds, which are relict environments within the study area and submitted to a degree of fragmentation comparatively much higher than that experienced by the habitats occupied by the esparto grasshopper (Ortego et al. 2010). In contrast with other studies on orthopterans, we did not find any genetic signature of sex-biased dispersal in the esparto grasshopper (Bailey et al. 2007; Ortego et al. 2011; Kindler et al. 2012). Thus, the fact that this species has a relatively high flying capacity (J. Ortego and P. J. Cordero, unpublished data) and that both sexes disperse from their natal areas at a similar rates may have contributed to increase gene flow and resulted in subtle genetic structures at the landscape scale here analysed (Ortego et al. 2011).

Despite the high potential for gene flow in this species and the shallow patterns of genetic structure above described, the dispersal routes of the species have been constrained by the spatial configuration of remnant semi-natural habitat patches within a matrix of land extensively devoted to agriculture. Landscape genetic analyses based on circuit theory showed that agricultural land offers ∽1000 times more resistance to gene flow than semi-natural habitats. The model exclusively including geographical distances between populations (i.e. IBD) was not significant and model fit increased with higher ratios of agricultural land/natural habitat resistance (Fig.5), indicating that the subtle genetic differentiation observed within the study area probably reflects the impact of farming and land clearing on the species' demographic dynamics. However, agricultural land cannot be considered an absolute barrier to dispersal in this species, as many of the semi-natural habitat patches where our populations are located are highly isolated within a matrix of cultures (see Fig.1) (Coulon et al. 2006). Lack of interpatch dispersal through agricultural lands should have resulted in stronger patterns of population genetic differentiation than we actually found, particularly if we consider the long time elapsed since fragmentation occurred, the short generation time of the species (1 year) and the fact that some of the studied habitat patches are too small and cannot sustain population sizes sufficiently large to buffer the effects of genetic drift (Frankham 1996).

Analyses of genetic diversity have shown that the proportion of suitable habitat around sampling sites is positively associated with local levels of genetic diversity, suggesting that populations located in less fragmented habitat patches sustain higher effective population sizes (e.g. Levy et al. 2013; Méndez et al. 2014). Further, genetic diversity was negatively correlated with average genetic differentiation with all other populations, indicating that isolation and limited gene flow have also contributed to erode genetic variability in some populations (e.g. Wang et al. 2011). This suggests that effective population sizes of the studied populations are not above a threshold that prevents the loss of genetic diversity and/or that the high potential for gene flow suggested by the subtle patterns of genetic structure observed in this species is not sufficient to counterbalance the effects of genetic drift (Lange et al. 2010).

In a broader context, the results of this study illustrate the importance of analysing the genetic consequences of extensive habitat destruction in ubiquitous species with high potential for gene flow. Understanding the consequences of habitat fragmentation in widespread species can help to determine the ‘minimum’ impact of this process for the entire community of related species (Lange et al. 2010; Keller et al. 2013b). Our study indicates that even widespread species that can persist in small habitat patches and show considerable gene flow also suffer from genetic drift and loss of genetic diversity due to habitat fragmentation. Thus, ubiquitous species less prone to suffer the effects of habitat fragmentation can inform on the landscape-level demographic processes experienced by other formerly co-distributed species that may have already gone locally extinct and provide a late-warning signal of the genetic consequences of historical habitat fragmentation. For these reasons, information on pervasive species, generally disregarded in most conservation genetic studies (but see Lange et al. 2010; Keller et al. 2013b), can greatly contribute to define the ultimate genetic impacts of habitat fragmentation, establish management practices aimed to restore patch connectivity and evaluate the efficiency of conservation actions in many regions of the world that have historically experienced massive land clearing linked to agricultural practices.

## Conclusions and implications for conservation

Our study indicates that a few remnant semi-natural habitat patches within a chronically and extensively fragmented landscape act as functional corridors that facilitate interpopulation gene flow and shape local levels of genetic diversity in the esparto grasshopper. These results, in conjunction with those of previous studies, indicate that the impact of land clearing for agriculture on dispersal patterns and gene flow can strongly vary even among related organisms that are expected to similarly respond to habitat fragmentation (Lange et al. 2010; Keller et al. 2013a,b; Levy et al. 2013). The preservation of these semi-natural patches may be particularly important for species with limited dispersal capacity and/or showing preferences for some microhabitats more geographically restricted. The extraordinarily deep genetic structure previously reported for the highly specialist *M. wagneri* suggests that local extinctions are not likely to be compensated by recurrent recolonizations in this species (Ortego et al. 2010, 2012), a pattern that considerably differs from the remarkable gene flow and metapopulation dynamics characterizing the more widespread *R. hispanica* (present study). Thus, a general recommendation derived from both this and previous studies in the area would be implementing management practices aimed to promote the conservation of organisms that are ecologically dissimilar, but prioritizing those species that are dispersal-limited and more likely to benefit from increasing or maintaining population connectivity (Gaublomme et al. 2011; Keller et al. 2013b). Given that most lands are private properties devoted to agriculture, management should therefore focus on preserving existing natural habitat patches or enhancing dispersal through riparian corridors (Keller et al. 2013a). Although some habitats have been recently protected or proposed for protection in the study region, these initiatives have been up to now mostly focused on saline/hypersaline lagoons and low grounds of particular importance due to their unique plant and animal communities (e.g. Cirujano-Bracamonte and Medina-Domingo 2002; Cordero and Llorente 2008). However, less attention has been paid to other vulnerable habitats such as esparto grass formations that provide important ecological functions and contribute to maintain biodiversity in a disproportionate way, particularly if we consider the insignificant area that they represent within these extensively human-modified landscapes (Manning et al. 2006). These habitats, often perceived as unproductive lands with no economic value, are submitted to different sources of human disturbance, including indiscriminate ploughing and aerial insecticide spraying for pest management, misleading habitat restoration practices (e.g. non-native pine plantations), uncontrolled waste dumping, and recurrent vegetation damage caused by livestock and off-road driving, among others (P. J. Cordero and J. Ortego, personal observations). Thus, regional conservation policies aimed to avoid these practices together with environmental education activities to generate local people's awareness for the preservation of these remnants habitats would greatly contribute to their long-term conservation.

## Data accessibility

Data available from the Dryad Digital Repository: http://dx.doi.org/10.5061/dryad.nv5j5.

## Supporting Information

**Table S1.** Microsatellite loci used to genotype esparto grasshoppers (*Ramburiella hispanica*). **Table S2.** Model selection to assess the association between population genetic diversity and cover of suitable habitat, average genetic differentiation (*F*_ST_) with other populations, latitude and longitude. **Table S3.** Pair-wise population *F*_ST_ and *F*_ST_ corrected for null alleles. **Table S4.** Proportion of membership of the studied populations to each of the four genetic clusters identified by the Bayesian method implemented in the program Tess.

## References

[b1] Aguirre MP, Noguerales V, Cordero PJ, Ortego J (2014). Isolation and characterization of polymorphic microsatellites in the specialist grasshopper *Ramburiella hispanica* (Orthoptera: Acrididae). Conservation Genetics Resources.

[b2] Aljanabi SM, Martinez I (1997). Universal and rapid salt-extraction of high quality genomic DNA for PCR-based techniques. Nucleic Acids Research.

[b3] Andrew RL, Ostevik KL, Ebert DP, Rieseberg LH (2012). Adaptation with gene flow across the landscape in a dune sunflower. Molecular Ecology.

[b4] Bacles CFE, Burczyk J, Lowe AJ, Ennos RA (2005). Historical and contemporary mating patterns in remnant populations of the forest tree *Fraxinus excelsior* L. Evolution.

[b5] Bailey NW, Gwynne DT, Ritchie MG (2007). Dispersal differences predict population genetic structure in Mormon crickets. Molecular Ecology.

[b6] Blanchet E, Lecoq M, Sword GA, Berthier K, Pages C, Billot C, Rivallan R (2012a). A comparative analysis of fine-scale genetic structure in three closely related syntopic species of the grasshopper genus *Calliptamus*. Canadian Journal of Zoology-Revue Canadienne de Zoologie.

[b7] Blanchet E, Lecoq M, Sword GA, Pages C, Blondin L, Billot C, Rivallan R (2012b). Population structures of three *Calliptamus* spp. (Orthoptera: Acrididae) across the Western Mediterranean Basin. European Journal of Entomology.

[b8] Blanchet S, Rey O, Etienne R, Lek S, Loot G (2010). Species-specific responses to landscape fragmentation: implications for management strategies. Evolutionary Applications.

[b9] Blondel J, Aronson J (1999). Biology and Wildlife of the Mediterranean Region.

[b10] Burnham KP, Anderson DR (1998). Model Selection and Inference: A Practical Information-Theoretic Approach.

[b11] Burnham KP, Anderson DR (2002). Model Selection and Multi-Model Inference: A Practical Information-Theoretic Approach.

[b12] Cordero PJ, Llorente V (2008). New data on the ‘silver-bell cricket’ (Orthoptera, Gryllidae), a forgotten and overlooked cricket subject to a high risk of extinction in western Europe. Graellsia.

[b13] Coulon A, Guillot G, Cosson JF, Angibault JMA, Aulagnier S, Cargnelutti B, Galan M (2006). Genetic structure is influenced by landscape features: empirical evidence from a roe deer population. Molecular Ecology.

[b14] Chapuis MP, Estoup A (2007). Microsatellite null alleles and estimation of population differentiation. Molecular Biology and Evolution.

[b15] Chapuis MP, Lecoq M, Michalakis Y, Loiseau A, Sword GA, Piry S, Estoup A (2008). Do outbreaks affect genetic population structure? A worldwide survey in *Locusta migratoria*, a pest plagued by microsatellite null alleles. Molecular Ecology.

[b16] Chen C, Durand E, Forbes F, Francois O (2007). Bayesian clustering algorithms ascertaining spatial population structure: a new computer program and a comparison study. Molecular Ecology Notes.

[b17] Cirujano-Bracamonte S, Medina-Domingo L (2002). Plantas acuáticas de las lagunas y humedales de Castilla-La Mancha.

[b18] DiBattista JD (2008). Patterns of genetic variation in anthropogenically impacted populations. Conservation Genetics.

[b19] DiLeo MF, Row JR, Lougheed SC (2010). Discordant patterns of population structure for two co-distributed snake species across a fragmented Ontario landscape. Diversity and Distributions.

[b20] Durand E, Jay F, Gaggiotti OE, Francois O (2009). Spatial inference of admixture proportions and secondary contact zones. Molecular Biology and Evolution.

[b21] Ersts PJ (2011). Geographic Distance Matrix Generator (Version 1.2.3).

[b22] Excoffier L, Laval G, Schneider S (2005). Arlequin ver. 3.0: an integrated software package for population genetics data analysis. Evolutionary Bioinformatics Online.

[b23] Fahrig L (2002). Effect of habitat fragmentation on the extinction threshold: a synthesis. Ecological Applications.

[b24] Fahrig L (2007). Non-optimal animal movement in human-altered landscapes. Functional Ecology.

[b25] Francois O, Durand E (2010). Spatially explicit Bayesian clustering models in population genetics. Molecular Ecology Resources.

[b26] Frankham R (1996). Relationship of genetic variation to population size in wildlife. Conservation Biology.

[b27] Frankham R (2005). Genetics and extinction. Biological Conservation.

[b28] Gaublomme E, Maebe K, van Doninck K, Dhuyvetter H, Li X, Desender K, Hendrickx F (2011). Loss of genetic diversity and increased genetic structuring in response to forest area reduction in a ground dwelling insect: a case study of the flightless carabid beetle *Carabus problematicus* (Coleoptera, Carabidae). Insect Conservation and Diversity.

[b29] Goudet J (1995). FSTAT (Version 1.2): a computer program to calculate F-statistics. Journal of Heredity.

[b30] Goudet J, Perrin N, Waser P (2002). Tests for sex-biased dispersal using bi-parentally inherited genetic markers. Molecular Ecology.

[b31] Guillot G (2009). On the inference of spatial structure from population genetics data. Bioinformatics.

[b32] Guo SW, Thompson EA (1992). A Monte Carlo method for combined segregation and linkage analysis. American Journal of Human Genetics.

[b33] Jakobsson M, Rosenberg NA (2007). CLUMPP: a cluster matching and permutation program for dealing with label switching and multimodality in analysis of population structure. Bioinformatics.

[b34] Jha S, Kremen C (2013). Urban land use limits regional bumble bee gene flow. Molecular Ecology.

[b35] Kalinowski ST (2005). HP-RARE 1.0: a computer program for performing rarefaction on measures of allelic richness. Molecular Ecology Notes.

[b36] Keller D, Holderegger R, van Strien MJ (2013a). Spatial scale affects landscape genetic analysis of a wetland grasshopper. Molecular Ecology.

[b37] Keller D, van Strien MJ, Herrmann M, Bolliger J, Edwards PJ, Ghazoul J, Holderegger R (2013b). Is functional connectivity in common grasshopper species affected by fragmentation in an agricultural landscape?. Agriculture Ecosystems & Environment.

[b38] Kindler E, Arlettaz R, Heckel G (2012). Deep phylogeographic divergence and cytonuclear discordance in the grasshopper *Oedaleus decorus*. Molecular Phylogenetics and Evolution.

[b39] Lange R, Durka W, Holzhauer SIJ, Wolters V, Diekotter T (2010). Differential threshold effects of habitat fragmentation on gene flow in two widespread species of bush crickets. Molecular Ecology.

[b40] Lee-Yaw JA, Davidson A, McRae BH, Green DM (2009). Do landscape processes predict phylogeographic patterns in the wood frog?. Molecular Ecology.

[b41] Levy E, Tomkins JL, LeBas NR, Kennington WJ (2013). Contrasting effects of landscape features on genetic structure in different geographic regions in the ornate dragon lizard, *Ctenophorus ornatus*. Molecular Ecology.

[b42] Lindenmayer DB, Fischer J (2006). Habitat Fragmentation and Landscape Change: An Ecological and Conservation Synthesis.

[b43] Manel S, Schwartz MK, Luikart G, Taberlet P (2003). Landscape genetics: combining landscape ecology and population genetics. Trends in Ecology & Evolution.

[b44] Manning AD, Fischer J, Lindenmayer DB (2006). Scattered trees are keystone structures – implications for conservation. Biological Conservation.

[b45] McRae BH (2006). Isolation by resistance. Evolution.

[b46] McRae BH, Beier P (2007). Circuit theory predicts gene flow in plant and animal populations. Proceedings of the National Academy of Sciences of the United States of America.

[b47] McRae BH, Dickson BG, Keitt TH, Shah VB (2008). Using circuit theory to model connectivity in ecology, evolution, and conservation. Ecology.

[b48] Méndez M, Vogeli M, Tella JL, Godoy JA (2014). Joint effects of population size and isolation on genetic erosion in fragmented populations: finding fragmentation thresholds for management. Evolutionary Applications.

[b49] Munshi-South J (2012). Urban landscape genetics: canopy cover predicts gene flow between white-footed mouse (*Peromyscus leucopus*) populations in New York City. Molecular Ecology.

[b50] Noss RF, Carroll CR, Csuti B, Meffe GK (1994). Habitat fragmentation. Principles of Conservation Biology.

[b51] Ortego J, Aguirre MP, Cordero PJ (2010). Population genetics of *Mioscirtus wagneri*, a grasshopper showing a highly fragmented distribution. Molecular Ecology.

[b52] Ortego J, Aguirre MP, Cordero PJ (2011). Fine-scale spatial genetic structure and within population male-biased gene-flow in the grasshopper *Mioscirtus wagneri*. Evolutionary Ecology.

[b53] Ortego J, Aguirre MP, Cordero PJ (2012). Landscape genetics of a specialized grasshopper inhabiting highly fragmented habitats: a role for spatial scale. Diversity and Distributions.

[b54] Pardo JE, Gómez R (1995). Orthopteroidea de los sistemas montañosos de Castilla-La Mancha (España). 3. Caelifera. Anales de Biologia (Murcia).

[b55] Pavlacky DC, Goldizen AW, Prentis PJ, Nicholls JA, Lowe AJ (2009). A landscape genetics approach for quantifying the relative influence of historic and contemporary habitat heterogeneity on the genetic connectivity of a rainforest bird. Molecular Ecology.

[b56] Pavlova A, Amos JN, Goretskaia MI, Beme IR, Buchanan KL, Takeuchi N, Radford JQ (2012). Genes and song: genetic and social connections in fragmented habitat in a woodland bird with limited dispersal. Ecology.

[b57] Phillipsen IC, Kirk EH, Bogan MT, Mims MC, Olden JD, Lytle DA (2015). Dispersal ability and habitat requirements determine landscape-level genetic patterns in desert aquatic insects. Molecular Ecology.

[b58] Quéméré E, Crouau-Roy B, Rabarivola C, Louis EE, Chikhi L (2010). Landscape genetics of an endangered lemur (*Propithecus tattersalli*) within its entire fragmented range. Molecular Ecology.

[b59] R Core Team (2012). R: A Language and Environment for Statistical Computing.

[b60] Rice WR (1989). Analyzing tables of statistical tests. Evolution.

[b61] Rosenberg NA (2004). DISTRUCT: a program for the graphical display of population structure. Molecular Ecology Notes.

[b62] Ruiz-González A, Gurrutxaga M, Cushman SA, Madeira MJ, Randi E, Gómez-Moliner BJ (2014). Landscape genetics for the empirical assessment of resistance surfaces: the European pine marten (*Martes martes*) as a target-species of a regional ecological network. PLoS One.

[b63] Saccheri I, Kuussaari M, Kankare M, Vikman P, Fortelius W, Hanski I (1998). Inbreeding and extinction in a butterfly metapopulation. Nature.

[b64] Segelbacher G, Cushman SA, Epperson BK, Fortin MJ, Francois O, Hardy OJ, Holderegger R (2010). Applications of landscape genetics in conservation biology: concepts and challenges. Conservation Genetics.

[b65] Seymour M, Rasanen K, Holderegger R, Kristjansson BK (2013). Connectivity in a pond system influences migration and genetic structure in threespine stickleback. Ecology and Evolution.

[b66] Storfer A, Murphy MA, Evans JS, Goldberg CS, Robinson S, Spear SF, Dezzani R (2007). Putting the ‘landscape’ in landscape genetics. Heredity.

[b67] Storfer A, Murphy MA, Spear SF, Holderegger R, Waits LP (2010). Landscape genetics: where are we now?. Molecular Ecology.

[b68] Wang IJ (2013). Examining the full effects of landscape heterogeneity on spatial genetic variation: a multiple matrix regression approach for quantifying geographic and ecological isolation. Evolution.

[b69] Wang IJ, Johnson JR, Johnson BB, Shaffer HB (2011). Effective population size is strongly correlated with breeding pond size in the endangered California tiger salamander, *Ambystoma californiense*. Conservation Genetics.

[b70] Wiesner KR, Loxdale HD, Kohler G, Schneider ARR, Tiedemann R, Weisser WW (2011). Patterns of local and regional genetic structuring in the meadow grasshopper, *Chorthippus parallelus* (Orthoptera: Acrididae), in Central Germany revealed using microsatellite markers. Biological Journal of the Linnean Society.

[b71] Zellmer AJ, Knowles LL (2009). Disentangling the effects of historic vs. contemporary landscape structure on population genetic divergence. Molecular Ecology.

