# Supplementary material for: Consequences of extensive habitat fragmentation in landscape-level patterns of genetic diversity and structure in the Mediterranean esparto grasshopper
Source: Evol Appl. 2015 Jun 1;8(6):621–32. doi: 10.1111/eva.12273 (PMC4479516; doi:10.1111/eva.12273)
Supplement: Supplementary file 1 [file eva0008-0621-sd1.docx]

Supporting Information

**Table S1.** Microsatellite loci used to genotype esparto grasshoppers (*Ramburiella hispanica*): annealing temperature (T_a_, in °C), number of alleles (*A*), expected heterozygosity (*H*_E_), observed heterozygosity (*H*_O_) and frequency of null alleles (NA) for each locus.

| Locus | T_a_ | *A* | *H*_E_ | *H*_O_ | NA |
| --- | --- | --- | --- | --- | --- |
| RhA105 | 55 | 30 | 0.932 | 0.651 | 0.20 |
| RhA108 | 55 | 32 | 0.935 | 0.882 | 0.07 |
| RhA112 | 55 | 40 | 0.964 | 0.646 | 0.27 |
| RhA113* | 55 | 31 | 0.945 | 0.383 | 0.30 |
| RhB107 | 55 | 16 | 0.899 | 0.885 | 0.06 |
| RhC1* | 60 | 17 | 0.898 | 0.422 | 0.37 |
| RhA2 | 55 | 25 | 0.930 | 0.445 | 0.29 |
| RhC2 | 55 | 19 | 0.874 | 0.563 | 0.24 |
| RhC112 | 55 | 33 | 0.941 | 0.908 | 0.04 |
| RhC113 | 55 | 18 | 0.901 | 0.800 | 0.11 |
| RhD2 | 55 | 14 | 0.828 | 0.762 | 0.06 |
| RhB2 | 55 | 12 | 0.793 | 0.796 | 0.08 |

* These loci deviated from Hardy-Weinberg equilibrium across all populations and were discarded for further analyses

**Table S2.** Model selection to assess the association between population genetic diversity [estimated as allelic richness (*A*_R_) and gene diversity (*H*_E_)] and cover of suitable habitat (A), average genetic differentiation (*F*_ST_) with other populations (B), latitude (C) and longitude (D).

| Model no. | Model | *K* | AIC_c_ | ∆AIC_c_ | ω*i* |
| --- | --- | --- | --- | --- | --- |
|  |  |  |  |  |  |
| (a) Allelic richness (*A*_R_) | |  |  |  |  |
| 1 | A+B | 4 | 25.21 | 0.00 | 0.51 |
| 2 | A+B+D | 5 | 27.57 | 2.37 | 0.16 |
| 3 | B | 3 | 27.77 | 2.56 | 0.14 |
| 4 | A+B+C | 5 | 29.12 | 3.91 | 0.07 |
| 5 | A+B+C+D | 6 | 30.68 | 5.47 | 0.03 |
| 6 | B+D | 4 | 30.78 | 5.57 | 0.03 |
| 7 | B+C | 4 | 30.79 | 5.59 | 0.03 |
| 8 | A | 3 | 33.59 | 8.39 | 0.01 |
| 9 | A+C | 4 | 33.81 | 8.60 | 0.01 |
| 10 | B+C+D | 5 | 34.65 | 9.45 | 0.00 |
| 11 | A+C+D | 5 | 35.42 | 10.21 | 0.00 |
| 12 | A+D | 4 | 36.87 | 11.67 | 0.00 |
| 13 | D | 3 | 38.41 | 13.20 | 0.00 |
| 14 | C | 3 | 38.54 | 13.34 | 0.00 |
| 15 | C+D | 4 | 41.66 | 16.45 | 0.00 |
|  |  |  |  |  |  |
| (b) Gene diversity (*H*_E_) | |  |  |  |  |
| 1 | A+B | 4 | -111.54 | 0.00 | 0.41 |
| 2 | A+B+D | 5 | -111.31 | 0.22 | 0.37 |
| 3 | A+B+C | 5 | -108.16 | 3.37 | 0.08 |
| 4 | A+B+C+D | 6 | -107.68 | 3.86 | 0.06 |
| 5 | A+C | 4 | -106.63 | 4.91 | 0.04 |
| 6 | B | 3 | -104.91 | 6.63 | 0.01 |
| 7 | A | 3 | -104.75 | 6.79 | 0.01 |
| 8 | A+C+D | 5 | -104.49 | 7.04 | 0.01 |
| 9 | B+D | 4 | -101.97 | 9.57 | 0.00 |
| 10 | A+D | 4 | -101.97 | 9.57 | 0.00 |
| 11 | B+C | 4 | -101.63 | 9.91 | 0.00 |
| 12 | B+C+D | 5 | -98.12 | 13.42 | 0.00 |
| 13 | D | 3 | -96.60 | 14.94 | 0.00 |
| 14 | C | 3 | -96.59 | 14.95 | 0.00 |
| 15 | C+D | 4 | -93.23 | 18.30 | 0.00 |

*K*, number of parameters in the model; AIC_c_, corrected Akaike’s information criterion (AIC) value; ∆AIC_c_, difference in AIC_c_ value from that of the strongest model; ω*i*, AIC_c_ weight.

**Table S3.** Pair-wise population *F*_ST_ (below the diagonal) and *F*_ST_ corrected for null alleles (above the diagonal). *F*_ST_ values in bold are statistically significant after sequential Bonferroni correction (*P* < 0.05).

|  |  | OCA | HUE | CER | ALT | LON | ALB | FAD | LAR | TIR | PEN | QUE | CAR | YEG | PAL | LAG | SCO | PED | ALC |
| --- | --- | --- | --- | --- | --- | --- | --- | --- | --- | --- | --- | --- | --- | --- | --- | --- | --- | --- | --- |
|  | OCA | - | 0.012 | 0.009 | 0.014 | 0.018 | 0.021 | 0.020 | 0.006 | 0.013 | 0.013 | 0.019 | 0.013 | 0.005 | 0.013 | 0.022 | 0.010 | 0.008 | 0.027 |
|  | HUE | **0.020** | - | 0.013 | 0.035 | 0.029 | 0.017 | 0.034 | 0.007 | 0.010 | 0.014 | 0.014 | 0.012 | 0.011 | 0.012 | 0.021 | 0.011 | 0.009 | 0.025 |
|  | CER | 0.012 | **0.017** | - | 0.016 | 0.026 | 0.015 | 0.028 | 0.011 | 0.005 | 0.013 | 0.013 | 0.018 | 0.011 | 0.011 | 0.017 | 0.017 | 0.011 | 0.017 |
|  | ALT | 0.019 | **0.040** | **0.019** | - | 0.025 | 0.014 | 0.028 | 0.012 | 0.013 | 0.012 | 0.020 | 0.019 | 0.021 | 0.017 | 0.026 | 0.019 | 0.016 | 0.030 |
|  | LON | **0.028** | **0.038** | **0.029** | **0.027** | - | 0.030 | 0.040 | 0.025 | 0.017 | 0.015 | 0.021 | 0.029 | 0.028 | 0.020 | 0.030 | 0.017 | 0.008 | 0.035 |
|  | ALB | 0.017 | **0.017** | 0.010 | 0.007 | **0.024** | - | 0.026 | 0.013 | 0.011 | 0.013 | 0.015 | 0.014 | 0.020 | 0.012 | 0.021 | 0.012 | 0.017 | 0.032 |
|  | FAD | **0.024** | **0.043** | **0.028** | **0.033** | **0.044** | **0.021** | - | 0.023 | 0.025 | 0.027 | 0.019 | 0.028 | 0.022 | 0.029 | 0.039 | 0.021 | 0.021 | 0.041 |
|  | LAR | 0.009 | 0.014 | 0.010 | 0.013 | **0.032** | 0.009 | **0.023** | - | 0.008 | 0.012 | 0.009 | 0.010 | 0.012 | 0.007 | 0.013 | 0.011 | 0.013 | 0.021 |
|  | TIR | **0.020** | 0.019 | 0.005 | 0.016 | **0.023** | 0.011 | **0.031** | 0.013 | - | 0.004 | 0.008 | 0.013 | 0.017 | 0.008 | 0.021 | 0.016 | 0.009 | 0.018 |
|  | PEN | 0.017 | **0.021** | 0.013 | 0.016 | 0.017 | 0.003 | **0.034** | 0.016 | 0.009 | - | 0.012 | 0.015 | 0.022 | 0.005 | 0.010 | 0.010 | 0.011 | 0.022 |
|  | QUE | **0.022** | **0.020** | 0.012 | **0.022** | **0.024** | 0.008 | **0.022** | 0.012 | **0.014** | 0.014 | - | 0.011 | 0.015 | 0.005 | 0.019 | 0.010 | 0.011 | 0.017 |
|  | CAR | **0.018** | 0.017 | **0.018** | **0.022** | **0.033** | 0.012 | **0.028** | 0.014 | **0.021** | 0.017 | 0.011 | - | 0.013 | 0.011 | 0.016 | 0.008 | 0.011 | 0.027 |
|  | YEG | 0.010 | **0.018** | 0.015 | **0.025** | **0.034** | **0.018** | **0.027** | 0.016 | 0.022 | **0.026** | **0.019** | 0.015 | - | 0.014 | 0.027 | 0.014 | 0.013 | 0.029 |
|  | PAL | 0.012 | 0.016 | 0.009 | 0.019 | **0.026** | 0.002 | **0.032** | 0.009 | 0.009 | 0.007 | 0.010 | 0.009 | 0.015 | - | 0.009 | 0.012 | 0.008 | 0.021 |
|  | LAG | 0.019 | **0.019** | 0.011 | **0.018** | **0.029** | 0.002 | **0.029** | 0.014 | 0.016 | 0.000 | 0.007 | 0.012 | **0.026** | 0.010 | - | 0.016 | 0.017 | 0.027 |
|  | SCO | 0.014 | **0.019** | **0.016** | **0.020** | 0.017 | 0.015 | **0.023** | 0.012 | **0.025** | 0.009 | 0.009 | 0.009 | 0.016 | 0.011 | 0.008 | - | 0.010 | 0.021 |
|  | PED | 0.014 | 0.011 | 0.016 | **0.017** | 0.011 | 0.015 | **0.023** | **0.019** | 0.012 | 0.006 | 0.009 | 0.011 | 0.019 | 0.007 | 0.013 | 0.013 | - | 0.008 |
|  | ALC | **0.036** | **0.032** | **0.021** | **0.034** | **0.041** | **0.032** | **0.047** | **0.030** | **0.023** | **0.027** | **0.019** | **0.030** | **0.038** | **0.025** | **0.025** | **0.025** | 0.013 | - |

**Table S4.** Proportion of membership of the studied populations to each of the four genetic clusters identified by the Bayesian method implemented in the program Tess.

| Locality | Code | Cluster 1 | Cluster 2 | Cluster 3 | Cluster 4 |
| --- | --- | --- | --- | --- | --- |
| Saladar de Ocaña | OCA | 0.053 | 0.786 | 0.007 | 0.154 |
| Huerta de Huerta | HUE | 0.039 | 0.779 | 0.006 | 0.176 |
| Laguna del Cerrillo | CER | 0.006 | 0.872 | 0.012 | 0.109 |
| Laguna del Altillo | ALT | 0.014 | 0.867 | 0.016 | 0.104 |
| Laguna de Longar | LON | 0.011 | 0.878 | 0.010 | 0.101 |
| Laguna de la Albardiosa | ALB | 0.007 | 0.876 | 0.006 | 0.111 |
| Villa de Don Fadrique | FAD | 0.004 | 0.887 | 0.007 | 0.101 |
| Laguna Larga | LAR | 0.007 | 0.833 | 0.005 | 0.155 |
| Laguna de Tírez | TIR | 0.004 | 0.826 | 0.003 | 0.167 |
| Laguna de Peña Hueca | PEN | 0.004 | 0.834 | 0.003 | 0.159 |
| Laguna de Quero | QUE | 0.004 | 0.838 | 0.005 | 0.153 |
| Laguna de Los Carros | CAR | 0.003 | 0.811 | 0.004 | 0.183 |
| Laguna de Las Yeguas | YEG | 0.004 | 0.674 | 0.006 | 0.317 |
| Laguna de Palomares | PAL | 0.005 | 0.881 | 0.011 | 0.103 |
| Laguna de La Laguna | LAG | 0.004 | 0.896 | 0.014 | 0.086 |
| Laguna de Salicor | SCO | 0.007 | 0.827 | 0.011 | 0.155 |
| Saladar de El Pedernoso | PED | 0.004 | 0.797 | 0.154 | 0.046 |
| Laguna de Alcahozo | ALC | 0.002 | 0.874 | 0.067 | 0.057 |
